# Microwave Assistant Synthesis, Antifungal Activity and DFT Theoretical Study of Some Novel 1,2,4-Triazole Derivatives Containing Pyridine Moiety

**DOI:** 10.3390/ijms15058075

**Published:** 2014-05-08

**Authors:** Guo-Xiang Sun, Ming-Yan Yang, Yan-Xia Shi, Zhao-Hui Sun, Xing-Hai Liu, Hong-Ke Wu, Bao-Ju Li, Yong-Gang Zhang

**Affiliations:** 1 School of Chemical and Biological Engineering, Yancheng Institute of Technology, Yancheng 224051, China; E-Mails: sunguoxiangyit@gmail.com (G.-X.S.); wuhk910@gmail.com (H.-K.W.); 2 College of Chemical Engineering, Zhejiang University of Technology, Hangzhou 310014, China; E-Mails: yangmingyanzjut@126.com (M.-Y.Y.); esunzhaohui@126.com (Z.-H.S.); 3 Institute of Vegetables and Flowers, Chinese Academy of Agricultural Sciences, Beijing 100014, China; E-Mail: shiyanxia813@163.com; 4 Biology Institute of Shandong Academy of Science, Jinan 250014, China

**Keywords:** 1,2,4-triazole, pyridine, synthesis, fungicidal activity, thioether, theoretical calculations

## Abstract

In order to investigate the biological activity of novel 1,2,4-triazole compounds, seventeen novel 1,2,4-triazole derivatives containing pyridine moiety were synthesized under microwave assistant condition by multi-step reactions. The structures were characterized by ^1^H NMR, MS and elemental analyses. The target compounds were evaluated for their fungicidal activities against *Stemphylium lycopersici* (Enjoji) Yamamoto, *Fusarium oxysporum.* sp. cucumebrium, and *Botrytis cinerea in vivo*, and the results indicated that some of the title compounds displayed excellent fungicidal activities. Theoretical calculation of the title compound was carried out with B3LYP/6-31G (*d*,*p*). The full geometry optimization was carried out using 6-31G (*d*,*p*) basis set, and the frontier orbital energy, atomic net charges were discussed, and the structure-activity relationship was also studied.

## 1. Introduction

**Figure 1.**
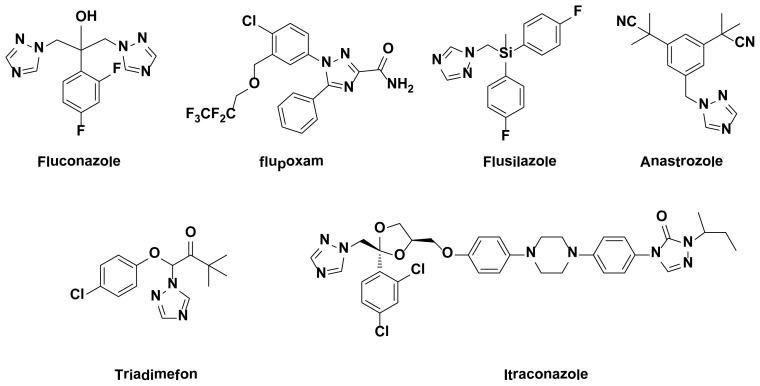
The commercial drug contain 1,2,4-triazole group.

In recent years, various nitrogen heterocyclic rings are known to exhibit interesting biological activities [1–9], such as triazole derivatives, thiazole derivatives, oxazole derivatives, and so on. 1,2,4-Triazole rings are typically planar aromatic systems, featuring an extensive chemistry [10,11]. 1,2,4-Triazole derivatives represent one of the most biologically active classes of heterocyclic compounds, and their derivatives are characterized with a broad spectrum of biological activity, including anti-cancer activity [12], antiparasitic activity [13], larvicidal activity[14], antifungal activity [15], herbicidal activity [16,17], antioxidant activity [18], cytostatic activity [19], brassinosteroid biosynthesis inhibitors [20], antimicrobial activity [21], and so on. Some compounds had been developed as commercial fungicides or herbicides (Figure 1), such as Triadimefon, Triadimenol, Flusilazole, Flupoxamand, and so on.

On the other hand, sulfur-linked 1,2,4-triazoles represent an important group of sulfur compounds that are promising for use in lead compound discovery, especially thione-substituted 1,2,4-triazole ring. Thus far, many bioactive sulfur-linked 1,2,4-triazole have been reported, such as antibacterial activity [22,23], antitumor activity [24], anti-HIV activity [25], anti-TMV (tobacco mosaic virus) activity [26], and so on.

**Figure 2.**
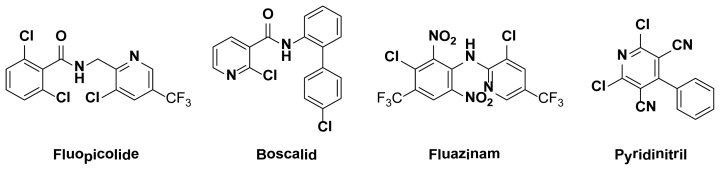
The commercial drug contain pyridine group.

Furthermore, pyridine is an important class of heterocyclic ring, which have attracted attention in past few years. The pyridine nucleus exists in numerous natural products and is extremely important in the chemistry of biological systems, such as antioxidant activity [27], antimicrobial activity [28], acetylcholinesterase inhibitors [29], antibacterial activity [30,31], anticancer activity [32,33], anti-HBV activity [34], and so on. In the fungicidal activity area, some pyridine derivatives can prevent *Ralstonia solanacearum* [35], *Cerospora beticola sacc.* [36], *Colletotrichum orbiculare* [37], and so on. Currently, some pyridine compounds have been developed and commercialized, for example, Fluopicolid, Boscalid, Fluazinam, and Pyridinitril (Figure 2).

There are many reports about each of the two heterocycles, but the combination of the pyridine ring with the 1,2,4-triazole ring in one molecule is seldom reported both in chemistry and their biological activity studies. In view of the facts mentioned above, and also as a part of our work [38–40] on the synthesis of bioactive lead compounds, the title compounds were designed by introducing the pyridine ring pharmacophore into the 1,2,4-triazole scaffold. Twenty-one novel 1,2,4-triazole derivatives were synthesized and characterized by ^1^H NMR, MS and elemental analysis. The antifungal activities of these compounds were tested *in vivo.*

## 2. Results and Discussion

### 2.1. Chemistry

**Scheme 1.**
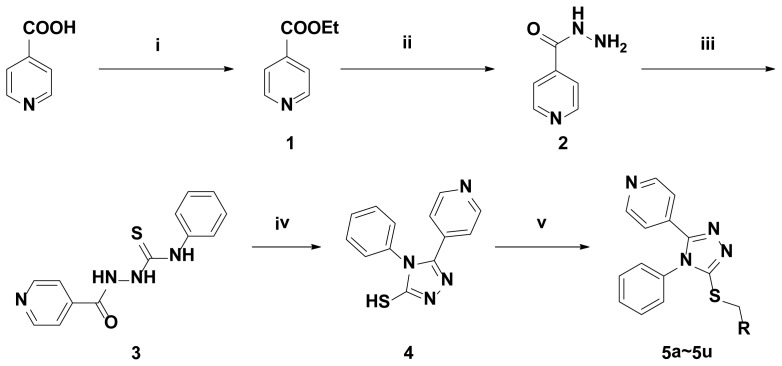
The synthetic route of title compounds. Reagents and Conditions: **i**, conc. H_2_SO_4_, EtOH, reflux; **ii**, NH_2_NH_2_H_2_O, EtOH, reflux; **iii**, Isothiocyanatobenzene, EtOH, reflux; **iv**, 1, NaOH, reflux, 2, HCl; **v**, RCl, NaOH, DMF, microwave (MW); **5a**, R = 2,4-Cl_2_Ph; **5b**, R = 3-CNPh; **5c**, R = 2-FPh; **5d**, R = 4-CNPh; **5e**, R = 2-Cl-5-Py; **5f**, R = 3-ClPh; **5g**, R = 4-BuPh; **5h**, R = 4-OCH_3_Ph; **5i**, R = 3,4-Cl_2_Ph; **5j**, R = 2-ClPh; **5k**, R = 4-BrPh; **5l**, R = 4-ClPh; **5m**, R = Propyl; **5n**, R = 2-Cl-thiazole-5-yl; **5o**, R = CN; **5p**, R = ((*E*)-methyl-2-(methoxyimino)-2-phenyl) acetate; **5q**, R = 3-FPh; **5r**, R = 2-MePh; **5s**, R = decyl; **5t**, R = vinyl; **5u**, R = ethyl acetate.

The synthesis procedures for title compounds were shown in Scheme 1. The intermediate **1** was synthesized easily from isonicotinic acid and alcohol under the concentrate, H_2_SO_4_, at refluxing condition. In addition, the intermediates **1** can be synthesized with high yield using SOCl_2_ or POCl_3_ as dehydrating agent. The intermediate **2** was easily given from ethyl isonicotinate and 85% hydrazine hydrate. The intermediate **3** can be obtained from the intermediate **2** and isothiocyanatobenzene. The intermediate **3** was easily cyclized to give intermediate **4** under alkaline condition, such as NaOH or KOH. The intermediate **4** is reacted with substituted benzyl chloride or alkyl chloride to afford compound **5**. The microwave irradiation assistant synthesis and conventional method was also employed in this experiment. NaOH/DMF/H_2_O system was applied under microwave irradiation. The best reaction condition is at 90 °C for 15 min under microwave irradiation synthesis. The yield is higher than that of conventional methods, and, in addition, the reaction time is shorter (Table 1).

The signal of CH_2_ protons of thioether, neighboring the triazole ring, was observed at δ 2.23–4.67 ppm, respectively. The chemical shifts of pyridine are divided into two double peaks. The ESI (electrospray ionization)-MS spectrum showed that the *m*/*z* of molecular ion, in accordance with its molecular formula. The elemental analysis result is in accordance with the calculated results.

### 2.2. Fungicidal Activities and Structure–Activity Relationship (SAR)

The *in vivo* fungicidal results of title compounds against *Stemphylium lycopersici* (Enjoji) Yamamoto, *Fusarium oxysporum.* sp. cucumebrium, and *Botrytis cinerea* were listed in Table 2, Zhongshengmycin, thiophanate-methyl, and cyprodinil were used as a control. As shown in Table 2, many of the title compounds showed good control efficacy against *Stemphylium lycopersici* (Enjoji) Yamamoto at a concentration of 500 μg/mL, only compound **5b** exhibited weak activity (5.21%) against *Stemphylium lycopersici* (Enjoji) Yamamoto. Notably, when the R is the electron donating group at the para position of the benzene ring, it showed good activity against *Stemphylium lycopersici* (Enjoji) Yamamoto. For example, compound **5g** (71.43%) and **5h** (85.12%) displayed excellent inhibition against *Stemphylium lycopersici* (Enjoji) Yamamoto, respectively. In addition, for the alkyl substituted compounds, the longer chain, the lower the activity. The order is C_11_H_23_ < C_4_H_9_ < CH_2_CN < CH_2_COOEt < CH=CH_2_. All the title compounds exhibited moderate inhibition effect against *Fusarium oxysporum.* sp. cucumebrium and the fungicidal activities range is 48%–66%. Surprisingly, all these compounds showed weak activity against *Botrytis cinerea*.

### 2.3. Molecular Total Energies and Frontier Orbital Energy Analysis

Molecular total energy and frontier orbital energy levels are listed in Table 3. The energy gap between HOMO and LUMO was calculated by B3LYP.

**Figure 3.**
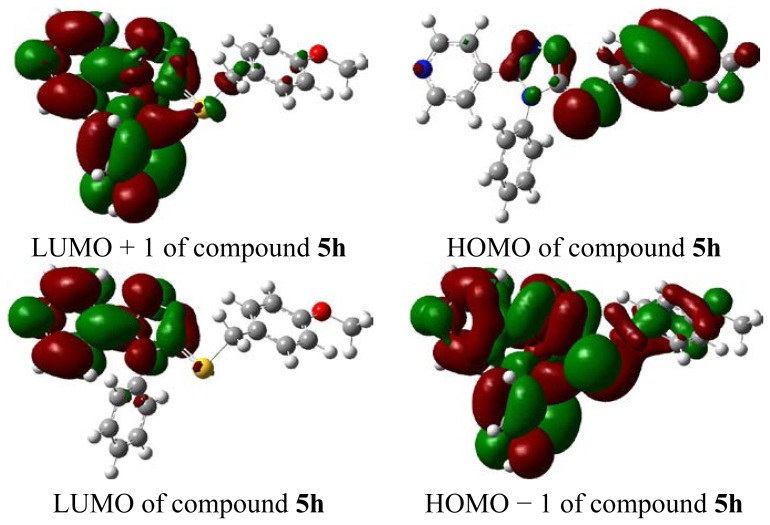
The HOMO (highest occupied molecular orbital) and LUMO (lowest unoccupied molecular orbital) compound **5h**.

According to the frontier molecular orbital theory, HOMO and LUMO are the most important factors that affect the bioactivity. HOMO has the priority to provide electrons, while LUMO can accept electrons first [41]. Thus, study on the frontier orbital energy can provide useful information about the biological mechanism. Taking the DFT (density functional theory) result for example, the geometry of the frame of the compound **5h** is hardly influenced by the introduction of, either the pyridine ring, 1,2,4-triazole ring, thioether group, or phenyl ring (Figure 3). The HOMO of the title compound is mainly located on the 4-OCH_3_ phenyl ring, 1,2,4-triazole ring, and thioether group. While, the LUMO of the title compound is located on the pyridine ring, 1,2,4-triazole ring, thioether group. The fact that the title compound has strong affinity suggests the importance of the frontier molecular orbital in the π–π stacking or hydrophobic interactions. This also implies that the orbital interaction between the title compound and the aromatic ring or some other side of residue chains of receptors is dominated by π–π or hydrophobic interaction among the frontier molecular orbitals.

### 2.4. Mulliken Atomic Charges and Electrostatic Potential Analyses

Table 4 exhibits the calculated Mulliken atomic charges except for atoms H. Taking DFT, for example, again, four atoms S18, C3, C23, and C27 are the most positively charged ones, which can interact with the negative charged part of the receptor easily. The negative charges are mainly located on atoms N1, N2, N4, N14, and O29, so they can interact easily with the positive part of the receptor. Therefore, we supposed this compound can combine the amino-acid residue on its surface by the interaction of the pyridine, triazole sulfur ether group, and OCH_3_ group, which may be responsible for the bioactivity.

**Figure 4.**
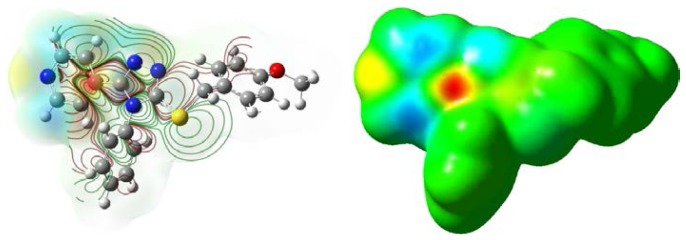
Electrostatic potential mapping on the electron density (*iso* value = 0.04).

Electrostatic potential of title compound was also calculated. From Figure 4, both oxygen atoms and nitrogen atoms have more negative charges. Perhaps the oxygen atoms and nitrogen atoms had some interaction with the receptor or acceptor.

## 3. Experimental Section

### 3.1. Materials and Reagents

All the reagents are of analytical grade or freshly prepared before use. The monitoring of the progress of all reactions and homogeneity of the synthesized compounds were carried out by thin layer chromatography (TLC), TLC analysis was performed on silica gel plate, which was obtained from Qingdao Ocean Chemicals (Qingdao, China). Melting points were determined using an X-4 apparatus (Taike, Beijing, China) and uncorrected (Taike, Beijing, China). ^1^H NMR spectra were measured on a Bruker AV 400 or 500 (Bruker, Fallanden, Switzerland) instrument using TMS as an internal standard and CDCl_3_ as the solvent. Mass spectra were recorded on a Thermo Finnigan LCQ Advantage LC/mass detector instrument (Thermo Finnigan, Waltham, MA, USA). Elemental analyses were performed on a Vario EL elemental analyzer (ELEMENTAR, Hanau, Germany). Microwave activation was carried out with CEM Discover™ focused microwave (2450 MHz, 300 W, CEM, Matthews, NC, USA).

### 3.2. Theoretical Calculations

On the basis of the above structure, an isolated molecule was selected as the initial structure, while DFT-B3LYP/6-31G (*d*,*p*) methods in Gaussian 03 package [42] were used to optimize the structure of the title compound. Vibration analysis showed that the optimized structures were in accordance with the minimum points on the potential energy surfaces. All the convergent precisions were the system default values, and all the calculations were carried out on a personal computer.

### 3.3. Chemical Synthesis

#### Synthesis of Intermediates

##### Ethyl Isonicotinate (**1**)

A mixture of isonicotinic acid (1.23 g, 10 mmol), 20 mL of ethanol, and 0.5 mL of H_2_SO_4_ were fluxed for 12 h in a 100 mL round-bottomed flask. After ethanol was evaporated under reduced pressure, about 20 mL of Na_2_CO_3_ solution (1 M) was added into the mixture. Then the mixture was extracted with ether. After evaporation of the solvent, the product **1** was obtained. Colorless liquid, yield: 90%.

##### Isonicotinyi Hydrazine (**2**)

A mixture of ethyl isonicotinate (1.44 g, 10 mmol) and 85% hydrazine hydrate (2 mL, 35 mmol) was heated under reflux for 6 h. The mixture was cooled to room temperature, filtered, washed with cool ethyl acetate, then dried to give white solid isonicotinyi hydrazine **2**. Yield (1.0 g, 73%).

##### 2-(4-Methyl-1, 2, 3-thiadiazole-5-carbonyl)-*N*-phenylhydrazinecarbothioamide (**3**)

A mixture of isonicotinyi hydrazine (1.37 g, 10 mmol) with isothiocyanatobenzene (1.35 g, 10 mmol) was refluxed for 5 h in ethanol. After cooling down to room temperature, the products were obtained and recrystallized from methanol to give **3**, yield 95%.

##### 5-Pyridyl-4-phenyl-4*H*-1,2,4-triazole-3-thiol (**4**)

A mixture of compound **3** (10 mmol) in aqueous NaOH solution (5 mL, 2 N) was refluxed for 4 h. After cooling down to room temperature, HCl aqueous solution (4 N) was added to afford a large amount of precipitate. The solid was filtered, dried and recrystallized from methanol to give intermediate **4**, yield 88%.

##### General Procedure for Thioether (**5**)

A CEM designed 10 mL pressure-rated vial was charged with DMF (5 mL), **4** (0.25 g, 1 mmol), RCH_2_Cl (1.1 mmol), and NaOH (0.05 g, 1.2 mmol). The mixture was irradiated in a CEM Discover Focused Synthesizer (150 w, 90 °C, 200 psi, 15 min). The mixture was cooled to room temperature by passing compressed air through the microwave cavity for 2 min. It was poured into cold ice (40 mL) and the formed precipitate was filtered. The crude solid was recrystallized from EtOH to give the title compounds **5a**. All the other compounds are synthesized according to the procedure.

##### 4-((5-(2,4-Dichlorobenzyl)thio)-4-phenyl-4*H*-1,2,4-triazol-3-yl)pyridine (**5a**)

White crystal, yield 83%, m.p. 184–186 °C; ^1^H NMR (CDCl_3_, 400 MHz), δ: 4.61 (s, 2H, SCH_2_), 7.17 (d, *J* = 7.5 Hz, 2H, Py), 7.20 (d, *J* = 8.3 Hz, 2H, Ph), 7.28–7.38 (m, 3H, ArH), 7.39 (s, 1H, ArH), 7.55–7.63 (m, 4H, ArH), 8.55 (d, *J* = 5.8 Hz, 2H, Py). MS (ESI), *m*/*z*: 414 (M + 1)^+^. Elemental analysis (%), calculated: C, 58.12; H, 3.41; N, 13.56; found: C, 58.22; H, 3.12; N, 13.43.

##### 3-(((4-Phenyl-5-(pyridin-4-yl)-4*H*-1,2,4-triazol-3-yl)thio)methyl)benzonitrile (**5b**)

White crystal, yield 90%, m.p. 179–181 °C; ^1^H NMR (CDCl_3_, 400 MHz), δ: 4.54 (s, 2H, SCH_2_), 7.16 (d, *J* = 7.1 Hz, 2H, Py), 7.30 (d, *J* = 6.0 Hz, 2H, ArH), 7.40 (t, *J* = 7.7 Hz, 1H, ArH), 7.52–7.59 (m, 4H, ArH), 7.67–7.69 (m, 2H, ArH), 8.55 (d, *J* = 5.5 Hz, 2H, Py). MS (ESI), *m*/*z*: 370 (M + 1)^+^. Elemental analysis (%), calculated: C, 68.27; H, 4.09; N, 18.96; found: C, 68.34; H, 3.98; N, 19.01.

##### 4-((5-(2-Fluorobenzyl)thio)-4-phenyl-4*H*-1,2,4-triazol-3-yl)pyridine (**5c**)

White crystal, yield 86%, m.p. 166–168 °C; ^1^H NMR (CDCl_3_, 400 MHz), δ: 4.57 (s, 2H, SCH_2_), 7.00–7.11 (m, 2H, Ar-H), 7.17 (d, *J* = 7.4 Hz, 2H, ArH), 7.27–7.32 (m, 3H, ArH), 7.51–7.57 (m, 4H, ArH), 8.55 (t, *J* = 6.1 Hz, 2H, Py). MS (ESI), *m*/*z*: 363 (M + 1)^+^. Elemental analysis (%), calculated: C, 66.28; H, 4.17; N, 15.46; found: C, 66.55; H, 4.25; N, 15.64.

##### 4-(((4-Phenyl-5-(pyridin-4-yl)-4*H*-1,2,4-triazol-3-yl)thio)methyl)benzonitrile (**5d**)

White crystal, yield 88%, m.p. 160–162 °C; ^1^H NMR (CDCl_3_, 400 MHz), δ: 4.56 (s, 2H, SCH_2_), 7.19 (d, *J* = 7.4 Hz, 2H, Py), 7.34 (d, *J* = 4.8 Hz, 2H, ArH), 7.56–7.63 (m, 7H, ArH), 8.56 (d, *J* = 4.9 Hz, 2H, Py). MS (ESI), *m*/*z*: 370 (M + 1)^+^. Elemental analysis (%), calculated: C, 68.27; H, 4.09; N, 18.96; found: C, 68.42; H, 4.13; N, 18.88.

##### 2-Chloro-5-(((4-phenyl-5-(pyridin-4-yl)-4*H*-1,2,4-triazol-3-yl)thio)methyl)pyridine (**5e**)

White crystal, yield 88%, m.p. 184–186 °C; ^1^H NMR (CDCl_3_, 400 MHz), δ: 4.51 (s, 2H, SCH_2_), 7.20 (d, *J* = 7.5 Hz, 2H, Py), 7.27 (d, *J* = 7.5 Hz, 1H, Py), 7.44 (d, *J* = 6.2 Hz, 2H, ArH), 7.56–7.63 (m, 3H, ArH), 7.82 (d, *J* = 8.2 Hz, 1H, Py), 8.40 (s, 1H, Py), 8.58 (t, *J* = 5.9 Hz, 2H, Py). MS (ESI), *m*/*z*: 381 (M + 1)^+^. Elemental analysis (%), calculated: C, 60.07; H, 3.71; N, 18.44; found: C, 59.99; H, 3.65; N, 18.58.

##### 4-(5-((3-Chlorobenzyl)thio)-4-phenyl-4*H*-1,2,4-triazol-3-yl)pyridine (**5f**)

White crystal, yield 84%, m.p. 153–155 °C; ^1^H NMR (CDCl_3_, 400 MHz), δ: 4.46 (s, 2H, SCH_2_), 7.15 (d, *J* = 7.6 Hz, 2H, Py), 7.21–7.36 (m, 6H, ArH), 7.52–7.60 (m, 3H, ArH), 8.55 (d, *J* = 4.3 Hz, 2H, Py). MS (ESI), *m*/*z*: 380 (M + 1)^+^. Elemental analysis (%), calculated: C, 63.40; H, 3.99; N, 14.79; found: C, 63.31; H, 3.78; N, 14.98.

##### 4-(5-((4-(*Tert*-butyl)benzyl)thio)-4-phenyl-4*H*-1,2,4-triazol-3-yl)pyridine (**5g**)

White crystal, yield 91%, m.p. 199–200 °C; ^1^H NMR (CDCl_3_, 400 MHz), δ: 1.31 (s, 9H, Bu), 4.52 (s, 2H, SCH_2_), 7.13 (d, *J* = 7.6 Hz, 2H, Py), 7.28–7.35 (m, 5H, ArH), 7.49–7.57 (m, 4H, ArH), 8.55 (d, *J* = 6.0 Hz, 2H, Py). MS (ESI), *m*/*z*: 401 (M + 1)^+^. Elemental analysis (%), calculated: C, 71.97; H, 6.04; N, 13.99; found: C, 72.21; H, 6.15; N, 14.21.

##### 4-(5-((4-Methoxybenzyl)thio)-4-phenyl-4*H*-1,2,4-triazol-3-yl)pyridine (**5h**)

White crystal, yield 89%, m.p. 114–116 °C; ^1^H NMR (CDCl_3_, 400 MHz), δ: 3.80 (s, 3H, OMe), 4.50 (s, 2H, SCH_2_), 6.83 (d, *J* = 8.6 Hz, 2H, ArH), 7.16 (d, *J* = 7.4 Hz, 2H, Py), 7.28–7.36 (m, 4H, ArH), 7.50–7.58 (m, 3H, ArH), 8.55 (d, *J* = 6.1 Hz, 2H, Py). MS (ESI), *m*/*z*: 375 (M + 1)^+^. Elemental analysis (%), calculated: C, 67.36; H, 4.85; N, 14.96; found: C, 67.44; H, 4.98; N, 14.87.

##### 4-(5-((3,4-Dichlorobenzyl)thio)-4-phenyl-4*H*-1,2,4-triazol-3-yl)pyridine (**5i**)

White crystal, yield 83%, m.p. 135–137 °C; ^1^H NMR (CDCl_3_, 400 MHz), δ: 4.50 (s, 2H, SCH_2_), 6.84 (d, *J* = 8.6 Hz, 2H, Ar-H), 7.17 (d, *J* = 7.4 Hz, 2H, Py), 7.28–7.31 (m, 3H, Ar-H), 7.50–7.58 (m, 3H, ArH), 8.54 (d, *J* = 6.0 Hz, 2H, Py). MS (ESI), *m*/*z*: 414 (M + 1)^+^. Elemental analysis (%), calculated: C, 58.12; H, 3.41; N, 13.56; found: C, 58.33; H, 3.12; N, 13.43.

##### 4-(5-((2-Chlorobenzyl)thio)-4-phenyl-4*H*-1,2,4-triazol-3-yl)pyridine (**5j**)

White crystal, yield 89%, m.p. 155–157 °C; ^1^H NMR (CDCl_3_, 400 MHz), δ: 4.65 (s, 2H, SCH_2_), 7.14 (d, *J* = 7.6 Hz, 2H, Py), 7.21–7.37 (m, 5H, ArH), 7.51–7.61 (m, 4H, ArH), 8.55 (d, *J* = 5.0 Hz, 2H, Py). MS (ESI), *m*/*z*: 380 (M + 1)^+^. Elemental analysis (%), calculated: C, 63.40; H, 3.99; N, 14.79; found: C, 63.63; H, 4.02; N, 14.88.

##### 4-(5-((4-Bromobenzyl)thio)-4-phenyl-4*H*-1,2,4-triazol-3-yl)pyridine (**5k**)

White crystal, yield 90%, m.p. 161–163 °C; ^1^H NMR (CDCl_3_, 400 MHz), δ: 4.48 (s, 2H, SCH_2_), 7.15 (d, *J =* 7.2 Hz, 2H, Py), 7.28–7.32 (m, 4H, ArH), 7.42 (d, *J =* 8.3 Hz, 2H, Ar-H), 7.51–7.60 (m, 3H, ArH). 8.60 (bs, 2H, Py). MS (ESI), *m*/*z*: 424 (M + 1)^+^. Elemental analysis (%), calculated: C, 56.74; H, 3.57; N, 13.23; found: C, 56.87; H, 3.67; N, 13.43.

##### 4-(5-((4-Chlorobenzyl)thio)-4-phenyl-4*H*-1,2,4-triazol-3-yl)pyridine (**5l**)

White crystal, yield 88%, m.p. 163 °C; ^1^H NMR (CDCl_3_, 400 MHz), δ: 4.50 (s, 2H, SCH_2_), 7.15 (d, *J =* 7.6 Hz, 2H, Py), 7.27–7.35 (m, 6H, ArH), 7.51–7.57 (m, 3H, Ar), 8.55 (d, *J =* 6.0 Hz, 2H, Py). MS (ESI), *m*/*z*: 380 (M + 1)^+^. Elemental analysis (%), calculated: C, 63.40; H, 3.99; N, 14.79; found: C, 63.51; H, 4.21; N, 14.65.

##### 4-(5-(Butylthio)-4-phenyl-4*H*-1,2,4-triazol-3-yl)pyridine (**5m**)

White crystal, yield 86%, m.p. 147–149 °C; ^1^H NMR (CDCl_3_, 400 MHz), δ: 0.94 (t, *J =* 7.4 Hz, 3H, CH_3_), 1.41–1.51 (m, 2H, CH_2_), 1.73–1.81 (m, 2H, CH_2_), 3.23 (t, *J =* 7.4 Hz, 2H, SCH_2_), 7.28–7.29 (m, 1H, Ar-H), 7.31 (d, *J =* 6.0 Hz, 2H, Py), 7.54–7.62 (m, 3H, ArH), 8.55 (d, *J =* 5.7 Hz, 2H, Py). MS (ESI), *m*/*z*: 380 (M + 1)^+^. Elemental analysis (%), calculated: C, 65.78; H, 5.84; N, 18.05; found: C, 65.87; H, 5.99; N, 17.98.

##### 2-Chloro-5-(((4-phenyl-5-(pyridin-4-yl)-4*H*-1,2,4-triazol-3-yl)thio)methyl)thiazole (**5n**)

White crystal, yield 88%, m.p. 199–202 °C; ^1^H NMR (CDCl_3_, 400 MHz), δ: 4.67 (s, 2H, SCH_2_), 7.22 (d, *J =* 7.3 Hz, 2H, Py), 7.38 (d, *J =* 6.2 Hz, 2H, Ar), 7.51 (s, H, thiazole-H), 7.56–7.64 (m, 3H, ArH), 8.55 (d, *J =* 6.0 Hz, 2H, Py). MS (ESI), *m*/*z*: 387 (M + 1)^+^. Elemental analysis (%), calculated: C, 52.91; H, 3.13; N, 18.15; found: C, 52.89; H, 3.12; N, 18.32.

##### 2-((4-Phenyl-5-(pyridin-4-yl)-4*H*-1,2,4-triazol-3-yl)thio)acetonitrile (**5o**)

White crystal, yield 79%, m.p. 237–240 °C; ^1^H NMR (CDCl_3_, 400 MHz), δ: 4.15 (s, 2H, SCH_2_), 7.30–7.31 (m, 4H, ArH and Py), 7.60–7.67 (m, 3H, ArH), 8.59 (d, *J =* 4.3 Hz, 2H, Py). MS (ESI), *m*/*z*: 294 (M + 1)^+^. Elemental analysis (%), calculated: C, 61.42; H, 3.78; N, 23.87; found: C, 61.38; H, 3.67; N, 23.98.

##### (*E*)-Methyl-2-(methoxyimino)-2-(2-(((4-phenyl-5-(pyridin-4-yl)-4*H*-1,2,4-triazol-3-yl)thio)methyl) phenyl)acetate (**5p**)

White crystal, yield 82%, m.p. 140–144 °C; ^1^H NMR (CDCl_3_, 400 MHz), δ: 3.61 (s, 3H, CH_3_), 3.78 (s, 3H, CH_3_), 4.48 (s, 2H, SCH_2_), 7.11–7.18 (m, 3H, ArH and Py), 7.27–7.32 (m, 4H, ArH), 7.47–7.56 (m, 4H, ArH), 8.56 (d, *J =* 4.6 Hz, 2H, Py). MS (ESI), *m*/*z*: 459 (M + 1)^+^. Elemental analysis (%), calculated: C, 65.48; H, 4.84; N, 12.22; found: C, 65.65; H, 5.01; N, 12.33.

##### 4-(5-((3-Fluorobenzyl)thio)-4-phenyl-4*H*-1,2,4-triazol-3-yl)pyridine (**5q**)

White crystal, yield 86%, m.p. 146–148 °C; ^1^H NMR (CDCl_3_, 400 MHz), δ: 4.52 (s, 2H, SCH_2_), 6.98 (t, *J =* 8.3 Hz, 1H, ArH), 7.12 (d, *J =* 9.6 Hz, 1H, ArH), 7.17 (d, *J =* 7.6 Hz, 2H, Py), 7.25–7.32 (m, 4H, ArH), 7.52–7.60 (m, 3H, ArH), 8.55 (d, *J =* 6.2 Hz, 2H, Py). MS (ESI), *m*/*z*: 363 (M + 1)^+^. Elemental analysis (%), calculated: C, 66.28; H, 4.17; N, 15.46; found: C, 66.42; H, 4.44; N, 15.64.

##### 4-(5-((2-Methylbenzyl)thio)-4-phenyl-4*H*-1,2,4-triazol-3-yl)pyridine (**5r**)

White crystal, yield 86%, m.p. 173–174 °C; ^1^H NMR (CDCl_3_, 400 MHz), δ: 2.35 (s, 3H, Me), 4.50 (s, 2H, SCH_2_), 7.10–7.17 (m, 4H, ArH and Py), 7.29–7.59 (m, 7H, ArH), 8.57 (d, *J =* 5.8 Hz, 2H, Py). MS (ESI), *m*/*z*: 359 (M + 1)^+^. Elemental analysis (%), calculated: C, 70.36; H, 5.06; N, 15.63; found: C, 70.15; H, 4.99; N, 15.76.

##### 4-(4-Phenyl-5-(undecylthio)-4*H*-1,2,4-triazol-3-yl)pyridine (**5s**)

White crystal, yield 84%, m.p. 82–84 °C; ^1^H NMR (CDCl_3_, 400 MHz), δ: 0.89 (t, *J =* 6.6 Hz, 3H, CH_3_), 1.24–1.45 (m, 16H, CH_2_), 1.74–1.82 (m, 2H, CH_2_), 3.32 (t, *J =* 7.3 Hz, 2H, SCH_2_), 7.28–7.33 (m, 4H, Ar-H and Py-H), 7.54–7.62 (m, 3H, ArH), 8.54 (d, *J =* 4.9 Hz, 2H, Py). MS (ESI), *m*/*z*: 409 (M + 1)^+^. Elemental analysis (%), calculated: C, 70.55; H, 7.89; N, 13.71; found: C, 70.80; H, 8.02; N, 13.56.

##### 4-(5-(Allylthio)-4-phenyl-4*H*-1,2,4-triazol-3-yl)pyridine (**5t**)

White crystal, yield 88%, m.p. 167–169 °C; ^1^H NMR (CDCl_3_, 400 MHz), δ: 3.95 (d, *J =* 7.1 Hz, 2H, SCH_2_), 5.19 (d, *J =* 10.0 Hz, 1H, =CH_2_), 5.34 (d, *J =* 15.9 Hz, 1H, =CH_2_), 5.94–6.00 (m, 1H, =CH), 7.27–7.33 (m, 4H, ArH and Py), 7.55–7.60 (m, 3H, ArH), 8.55 (d, *J =* 5.9 Hz, 2H, Py). MS (ESI), *m*/*z*: 295 (M + 1)^+^. Elemental analysis (%), calculated: C, 65.28; H, 4.79; N, 19.03; found: C, 65.42; H, 4.90; N, 19.09.

##### Ethyl-2-((4-phenyl-5-(pyridin-4-yl)-4*H*-1,2,4-triazol-3-yl)thio)acetate (**5u**)

White crystal, yield 92%, m.p. 162–164 °C; ^1^H NMR (CDCl_3_, 400 MHz), δ: 1.27 (t, *J =* 7.1 Hz, 3H, Me), 4.12 (s, 2H, SCH_2_), 4.22 (q, *J =* 7.1 Hz, 2H, CH_2_), 7.30–7.33 (m, 4H, ArH and Py), 7.55–7.60 (m, 3H, ArH), 8.55 (d, *J =* 4.6 Hz, 2H, Py). MS (ESI), *m*/*z*: 341 (M + 1)^+^. Elemental analysis (%), calculated: C, 59.98; H, 4.74; N, 16.46; found: C, 59.89; H, 4.99; N, 16.55.

### 3.4. Fungicidal Activities

Fungicidal activity of compounds **5a**–**5u** against *Stemphylium lycopersici* (Enjoji) Yamamoto*, Fusarium oxysporum.* sp. cucumebrium, and *Botrytis cinerea* were evaluated according to reference, and a potted plant test method was adopted. Germination was conducted by soaking cucumber seeds in water for 2 h at 50 °C and then keeping the seeds moist for 24 h at 28 °C in an incubator. When the radicles were 0.5 cm, the seeds were grown in plastic pots containing a 1:1 (*v*/*v*) mixture of vermiculite and peat. Cucumber and tomato plants used for inoculations were at the stage of two seed leaves. Tested compounds and commercial fungicides were sprayed with a hand sprayer on the surface of the seed leaves on a fine morning, at the standard concentration of 500 μg/mL, zhongshengmycin, thiophanate-methyl and cyprodinil were used as a control. After 2 h, inoculations of *Stemphylium lycopersici* (Enjoji) Yamamoto was carried out by spraying fungal suspension, inoculation of *Fusarium oxysporum.* sp. cucumebrium was carried out by spraying mycelial suspension, inoculation of *Botrytis cinerea* was carried out by radicle soaking. The experiment was repeated 4 times. After inoculation, the plants were maintained at 18–30 °C (mean temperature of 24 °C and above 80% relative humidity (RH)). The fungicidal activities were evaluated when the nontreated cucumber plant (blank) fully developed symptoms. The area of inoculated treated leaves covered by disease symptoms was assessed and compared to those of nontreated ones to determine the average disease index. The relative control efficacy of compounds compared to the blank assay was calculated via the following equation:

(1) Relative control efficacy (%)=(CK-PT)/CK×100%

where *CK* is the average disease index during the blank assay and *PT* is the average disease index after treatment during testing.

## 4. Conclusions

In summary, a series of 1,2,4-triazole derivatives were synthesized containing pyridine ring in good yields. The preliminary bioassays showed that some of the compounds had good fungicidal activity. The present findings provided a powerful complement to the SARs of fungicides, and warrant future investigation of the mechanism of action of these analogs.

## Figures and Tables

**Table 1.** Comparison of yields of **5a** through methods with or without microwave (MW) irradiation.

No.	Method	Time	Temperature/°C	Yield/%
**5a**	No-MW	24 h	r.t.	80
**5a**	No-MW	10 min	90	38
**5a**	MW	10 min	90	81
**5a**	MW	15 min	90	83
**5a**	MW	20 min	90	83

**Table 2.** The antifungal activity (%) of title compounds *in vivo* at 500 ppm.

No.	*Stemphylium lycopersici* (Enjoji) Yamamoto	*Fusarium oxysporum.* sp. cucumebrium	*Botrytis cinerea*
**5a**	53.57	66.67	24.44
**5b**	5.21	64.86	20.42
**5c**	50.30	65.56	28.89
**5d**	33.04	66.67	23.33
**5e**	43.15	64.44	24.44
**5f**	46.23	66.94	14.44
**5g**	71.43	63.93	20.00
**5h**	85.12	64.44	20.00
**5i**	73.51	63.33	16.67
**5j**	62.20	66.67	15.56
**5k**	42.86	64.44	6.67
**5l**	66.98	64.44	17.78
**5m**	54.17	64.81	26.67
**5n**	46.58	65.56	10.00
**5o**	65.67	48.89	13.33
**5p**	66.07	54.44	15.56
**5q**	67.86	53.33	22.22
**5r**	75.00	53.33	15.56
**5s**	51.79	53.33	12.22
**5t**	79.76	55.56	12.22
**5u**	75.89	48.89	20.00

Zhongshengmycin	59.58		
Thiophanate-Methyl		81.69	
Cyprodinil			45.56

**Table 3.** Total energy, frontier orbital energy. DFT, density functional theory; LUMO, lowest unoccupied molecular orbital; HOMO, highest occupied molecular orbital.

*E*	DFT
*E*_total_/Hartree b	−1503.11499092
*E*_HOMO_/Hartree	−0.21450
*E*_LUMO_/Hartree	−0.05529
Δ*E* a/Hartree	0.15921

a Δ*E*= *E*_LUMO_ − *E*_HOMO_;

b Hartree = 4.35974417 × 10^−18^, J = 27.2113845 eV.

**Table 4.** Mulliken atomic charges except for atoms H (e).

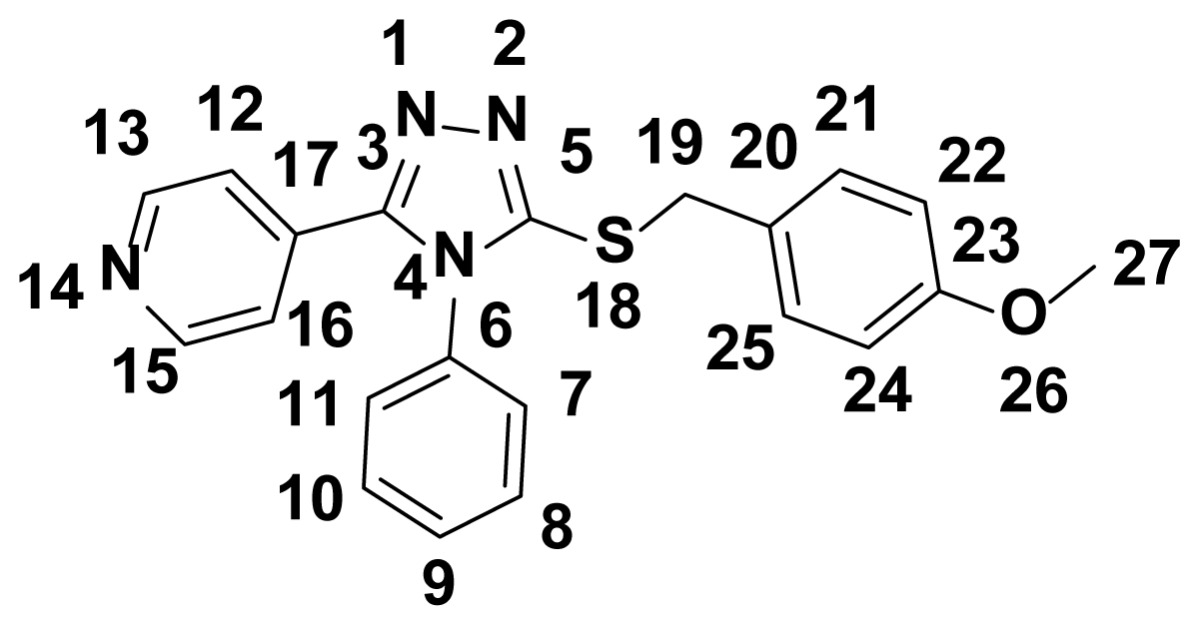	

Atom	Charge(DFT)
S18	0.326749
N14	−0.359494
N4	−0.757709
N2	−0.319626
N1	−0.308887
C16	0.035720
C15	0.125270
C13	0.135365
C12	0.056497
C17	0.127367
C3	0.361752
C5	0.175208
C6	0.170471
C7	0.092394
C8	−0.002097
C9	0.040403
C10	−0.001456
C11	0.093994
C19	−0.146321
C20	0.128847
C21	−0.032559
C22	−0.012577
C23	0.287098
C24	0.008964
C25	0.034697
O26	−0.557033
C27	0.296964
